# Laparoscopic management of an intramurally migrated intrauterine contraceptive device (IUCD) without serosal perforation: a case report

**DOI:** 10.1097/RC9.0000000000000970

**Published:** 2026-09-16

**Authors:** Mekyus E. Sanga, Daisy A. Mhando, Justin N. Ilunga, Daniel Michael, Peter Majinge

**Affiliations:** a Department of Obstetric and Gynaecology, Medical College, Aga Khan University, Dar es Salaam, Tanzania; b Department of Obstetric and Gynaecology, Comprehensive Community-Based Rehabilitation in Tanzania (CCBRT) – Hospital, Dar es Salaam, Tanzania

**Keywords:** case report, hysteroscopy, intramural migration, intrauterine contraceptive device (IUCD), laparoscopy, ultrasonography

## Abstract

**Introduction and importance:**

An intrauterine contraceptive device (IUCD) is a highly effective, long-term, and safe contraceptive method. However, serious complications, such as uterine perforation, can occur, particularly in high-risk women, thereby necessitating close follow-up.

**Case presentation:**

A 35-year-old woman, para 3 + 3, with 3 living children, all delivered by cesarean section, presented with persistent lower abdominal pain and vaginal bleeding 5 months postdelivery and IUCD insertion. The IUCD string was not visualized on either clinical examination or hysteroscopy. Ultrasonography suggested a migrated IUCD. Laparoscopy revealed an intramurally migrated IUCD, which was successfully removed, and she recovered well.

**Clinical discussion:**

Postpartum insertion of an IUCD increases the risk of displacement. When the IUCD string is not visualized, thorough evaluation is recommended for early diagnosis and management of this potentially serious complication.

**Conclusion:**

Routine follow-up after IUCD insertion is crucial, especially for high-risk women, to detect and manage migration early, with laparoscopy as the preferred treatment when feasible. However, laparotomy is still an optimal option in more complex cases.

## Introduction and importance

Intrauterine contraceptive device (IUCD) is a popular birth control method, valued for being highly effective, long-term, reversible, and safe. An estimated 14–27% of women worldwide use this method[1]. In Tanzania, this method is utilized by only 9% of women using artificial family planning, making it the least popular compared with implants, which are used by 14% of women[2]. However, despite its excellent safety profile, there is a risk of side effects or complications during and after insertion. This work has been reported in accordance with the SCARE guidelines[3].

Pain and irregular bleeding are the most common side effects and the most frequently cited reasons for premature removal, at 28–35% and 10–19%, respectively[4]. While uncommon, IUCD complications include device expulsion (3–6% within the first year), pelvic inflammatory disease (1% in the initial 20 days, declining to 0.5% by 3–6 months), contraceptive failure (0.1–0.6% in the first year, with elevated ectopic pregnancy risk if failure occurs), and uterine perforation (0.01%)^[^5–9^]^.

Uterine perforation, which can occur at the time of IUCD placement or slowly thereafter, is the most serious complication. In this report, we share a case of intramural migration of an IUCD following placement at cesarean delivery. The case was successfully managed laparoscopically. This case highlights the importance of scheduled follow-ups after IUCD insertion, especially for those at higher risk. Clinicians should maintain a high index of suspicion for serious complications in women presenting with persistent pain after IUCD insertion.

HIGHLIGHTS

- Intra-cesarean IUCD placement may increase the risk of positional abnormality compared with delayed insertion.
- Standardized follow-up after intra-cesarean IUCD placement is essential for the early detection of device migration and the prevention of subsequent injury.
- Laparoscopy serves as an effective, minimally invasive surgical option for managing transmural IUCD migration in appropriate clinical cases.

## Case presentation

We present S.R., a 35-year-old female of Black African ethnicity who is para 3 + 3 with 3 living children, all delivered by cesarean section at term. Her last delivery was on 2 June 2025, during which a copper IUCD was also inserted.

She presented with a history of intermittent lower abdominal pain associated with on-and-off vaginal bleeding that persisted after the puerperium. The pain was cramping in nature, non-radiating and of moderate severity. However, she had no fever, vomiting, or diarrhea and had normal micturition habits. The bleeding was small in amount, requiring changes of partially soaked pads at least 3 times a day, but there was no anemia; she also had no abnormal vaginal discharge.

Her past obstetric history is notable for a first-trimester miscarriage in 2018 and a cesarean delivery secondary to macrosomia in 2019. In 2020, she had two consecutive miscarriages, both in the first trimester. In 2021, she delivered by cesarean section secondary to a previous scar. Following her first delivery, she used a copper IUCD for contraception, which was spontaneously expelled after 2 weeks. She then used Implanon for 18 months but had it removed because of persistent amenorrhea throughout its use (details of her past obstetric history are summarized in Table 1).

**Table 1:** Timeline of events.

Timeline	Event
2018 (Age 28 years)	First-trimester miscarriage, which was surgically managed
2019 (Age 29 years)	Cesarean delivery secondary to macrosomia
2020 (Age 30 years)	Two consecutive first-trimester miscarriages, which were medically managed
2021 (Age 31 years)	Cesarean delivery secondary to previous scar
2025 (Age 35 years)	Cesarean delivery secondary to two previous scars, IUCD is placed intraoperatively
At presentation (5 months postdelivery)	Persistent lower abdominal pain and bleeding

On examination during the 6-week postnatal follow-up, she was alert, afebrile, not dyspneic, not pale, not jaundiced, and had no limb edema with stable vital signs. Her abdomen was of normal contour, with a healed Pfannenstiel incision scar, soft and mildly tender on deep palpation, but with no rebound tenderness. The IUCD string was not seen on speculum examination. This was attributed to its recent intraoperative placement, as the string might not have protruded yet. The patient was reassured and managed conservatively, and she was scheduled to return for follow-up.

During the follow-up visit, the symptoms had persisted, with slightly increased severity, prompting sonographic evaluation. The ultrasound report suggested a transversely positioned IUCD on the uterine fundus, a normal endometrial thickness with an empty cavity, and normal urinary bladder and adnexa. An impression of a malpositioned/displaced IUCD was made (Fig. 1).

**Figure 1.**
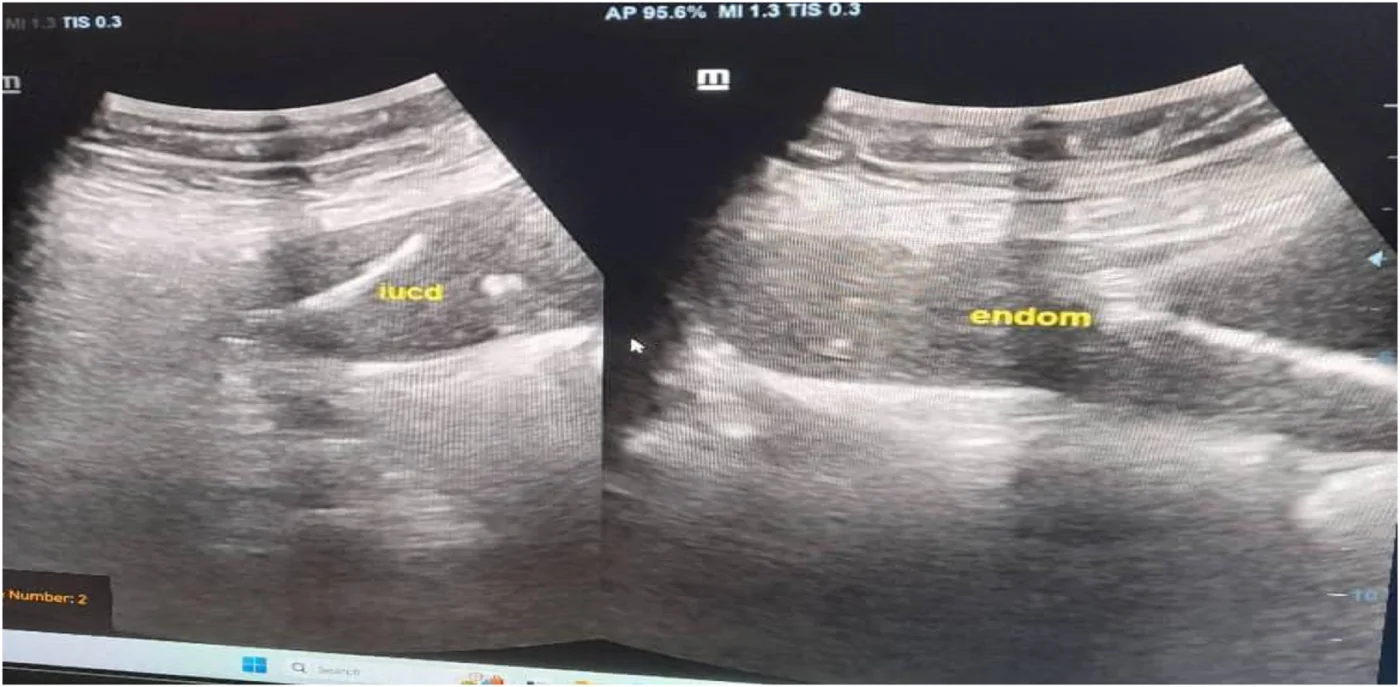
Ultrasound showing the IUCD located in the myometrium at the fundus (left) and an empty endometrium (right).

The patient was examined under anesthesia, and blind removal was attempted; however, the device could not be found. The patient was counseled and scheduled for hysteroscopic evaluation, which revealed an empty endometrial cavity, and the device could not be visualized. With these findings, it was suspected that the device was embedded in the myometrium or had perforated the uterus.

Laparoscopy was planned for exploration and removal, with counseling on the possibility of conversion to laparotomy. Intraoperatively, the tip of one arm of the device was visualized subserosally on the left uterine fundus near the cornu. The urinary bladder and lower anterior uterine wall were partially adherent to the anterior abdominal wall. Both adnexa were free and normal in appearance (Fig. 2).

**Figure 2.**
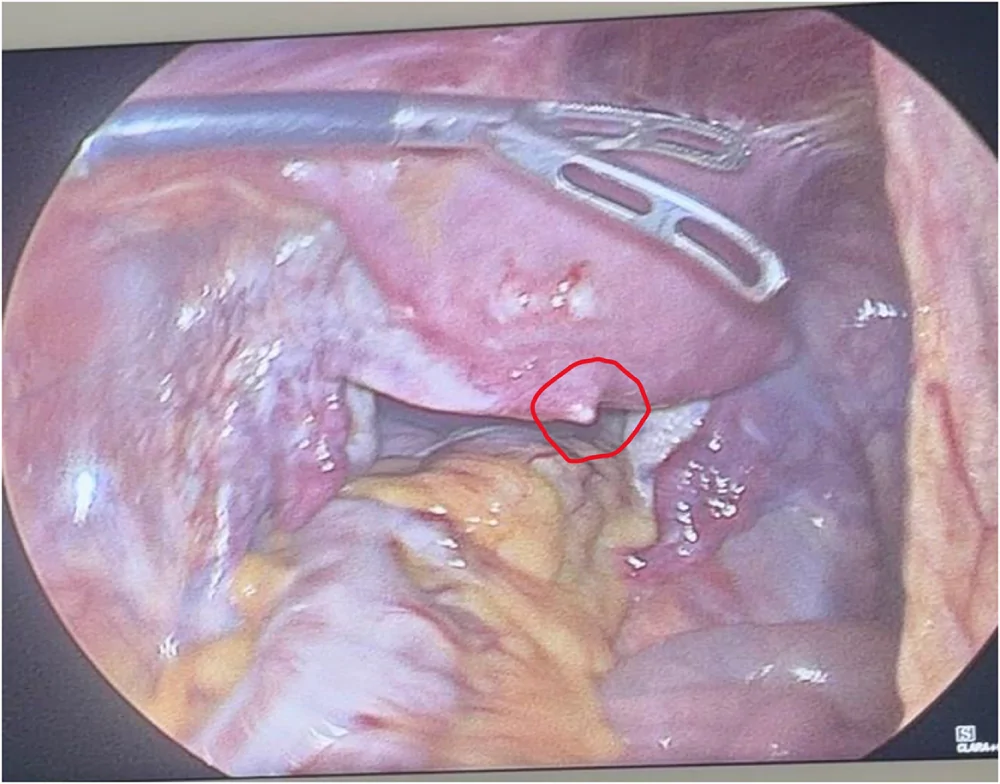
A laparoscopic image showing the tip of the IUCD on the left uterine fundus (red circle).

An incision was made in the left fundal region to further explore; an intact IUCD was removed successfully. The uterus was repaired with Vicryl 1 suture, and hemostasis was achieved (Fig. 3a and b). No complications occurred intra- and postoperatively.

**Figure 3.**
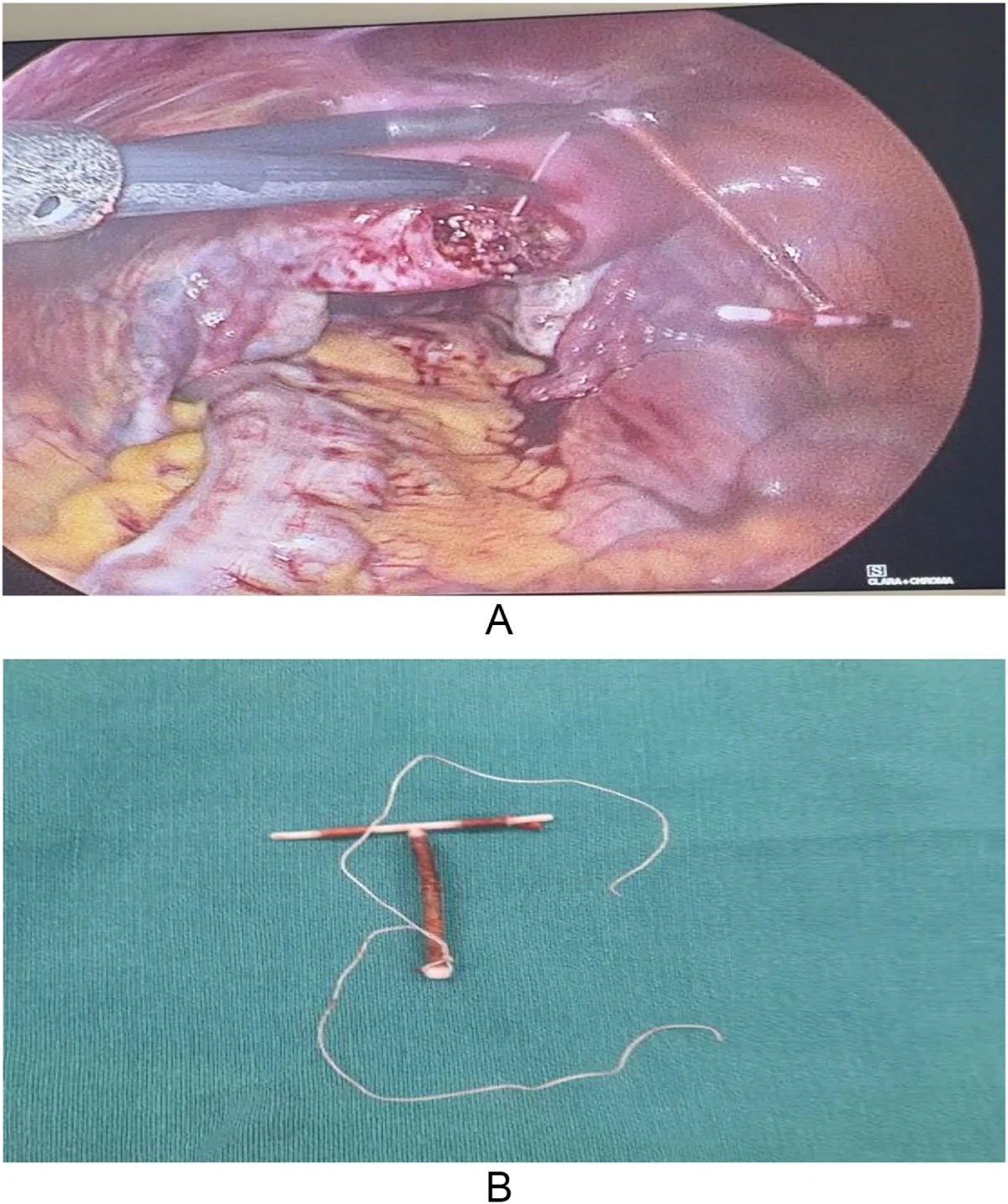
(a) Laparoscopic view of the intact copper IUCD in the final stage of removal; and (b) the intact copper IUCD after removal.

The patient recovered well and was discharged home the following day. At the time of discharge, the patient was counseled on alternative methods of contraception, and she opted for an implant. During the follow-up visit in the subsequent week, she reported no further pain or abnormal bleeding and had no new symptoms. At the 4-week follow-up, the laparoscopic port sites had completely healed, and she remained asymptomatic. The 3-month follow-up was uneventful.

## Clinical discussion

The IUCD is the most popular contraceptive method for long-term family planning. Significant complications are rare; however, intramural migration or perforation is the most feared complication and can be life-threatening. Its incidence ranges from 1/350 to 1/2500 insertions^[^6,10^]^. This can manifest in two ways: embedment, in which case it does not exceed the serosa, remaining at the level of the myometrium, as was found in this case, or perforation, in which it can migrate into organs within the peritoneal cavity, a situation encountered in 85% of cases[6]. In a recent systematic review of case reports and case series, the most common sites of migration are the intestine, bladder, and omentum[11].

Contrary to the present case, a previously reported case by Melinda *et al* found a migrated IUD at the uterine cervix, between the serosa and the lumbosacral plexus, causing chronic lower back pain; this case was also successfully managed laparoscopically[12].

Risk factors for this type of complication include insertion of an IUCD during the postpartum or post-abortion period, as occurred in this case, in which the device was placed during cesarean section. Other contributing factors are insertion by an inexperienced practitioner, multiparity, nulliparity, a scarred or immobile uterus, an extremely anteverted or retroverted uterus, discrepancy between the size of the IUCD and the uterine cavity, insertion technique, excessive force during insertion, and an atrophic uterus[11]. Additional reported risk factors include young age[12], a history of previous IUCD failure, and a distorted uterine cavity^[^13–15^]^.

The patient in the present case had a history of spontaneous IUCD expulsion; however, she had no known anatomical abnormalities, and the device was inserted by a senior obstetrician. Therefore, caution should be exercised during intra-cesarean IUCD placement to ensure accurate positioning and avoid the use of excessive force, even when the procedure is performed by experienced practitioners and in the absence of additional risk factors.

A transmurally migrated device can take a variable course; one review illustrates that the bowel is the most affected organ in cases of perforated and migrated IUCDs (intestine 32%, colon 4%, appendix 1%, ileum 5%, rectum 12%, sigmoid 9%)[16], and the second most affected organ is the bladder (24%), often associated with lithiasis and urinary symptoms^[^11,17–19^]^. Very rarely, the device can migrate to sites such as the subcutaneous tissue, creating a sinus tract[20].

The mechanism by which the device migrates is still a mystery; however, uterine contractions are the proposed mechanism[21]. Device migration can occur immediately at the time of insertion, most commonly during postpartum placement, as was the case here. It can also occur gradually over time through myometrial erosion[22]. In the present case, it cannot be stated with certainty which mechanism was responsible. However, in line with the initial mild symptoms together with a missing string at the 6-week follow-up, it is more likely that the migration occurred immediately.

Most of the time, perforation and migration are asymptomatic until a long time has elapsed, when they are identified incidentally[23] or present with symptoms of complications such as lower abdominal pain, ectopic pregnancy, or intractable urinary symptoms[24]. Because of these later complications, it is strongly recommended that a missing IUCD be thoroughly evaluated for migration before assuming it was spontaneously expelled. In addition, the World Health Organization (WHO) advocates that once a migrated IUCD is identified, it should be managed regardless of whether it is symptomatic or not^[^25,26^]^.

When the IUCD string is not visible at the external cervical os, imaging is advised to rule out uterine perforation or device migration[16]. Ultrasonography is the first-line investigation to confirm the position of the IUCD or to assess for a missing IUCD. Additional imaging such as plain abdominal X-ray, CT scan, or MRI may be required to further evaluate, especially in challenging or complex cases and associated complications, and to appropriately plan management[16]. For example, in one case of migration of an IUCD to the urinary bladder complicated by stone formation, the diagnosis was confirmed by plain abdominal radiography, and cystoscopy was used for management[19]. Boortz *et al* described radiological findings across different imaging modalities for migrated IUCDs[27]. Availability and accessibility of advanced imaging techniques are still challenging in most low-resource settings. In this case, only ultrasound was performed, and displacement of the device was identified.

The approach to retrieving a migrated IUCD depends on the device’s current location and the type of perforation involved. Management options include hysteroscopy for cases of partial perforation, colonoscopy if the device has migrated into the gastrointestinal lumen, cystoscopy for bladder migration, laparoscopy, and laparotomy[18]. Laparoscopy was used to confirm the diagnosis and successfully manage the present case.

## Conclusion

Routine follow-up after intra-cesarean IUCD insertion is important to enable early recognition and appropriate management of a displaced or migrated IUCD. This is especially important for women at higher risk of complications, such as those in the postpartum period or with multiple cesarean sections, which pose a risk of anatomical distortion. Minimally invasive procedures, such as laparoscopy, are the first-line management for a migrated IUCD, calling for improved availability and accessibility in resource-limited settings. However, laparotomy remains an optimal treatment option in more complex cases or when minimal access surgery is limited by other patient-specific factors.

## Data Availability

Not applicable.

## Patient perspective

I am pleased that my condition has been identified and successfully treated. I would like to express my sincere gratitude to the entire healthcare team for their care. Although I was initially anxious about returning to theatre, their thorough counseling alleviated my concerns, and I am now relieved to have made a full recovery.
